# Release of protein N-glycans by effectors of a Hofmann carboxamide rearrangement

**DOI:** 10.3389/fmolb.2022.983679

**Published:** 2022-09-12

**Authors:** Mumtaz Kasim, Malissa Schulz, Anja Griebel, Akshay Malhotra, Barbara Müller, Hans Henning von Horsten

**Affiliations:** ^1^ HTW Berlin-University of Applied Sciences, Life Science Engineering, Berlin, Germany; ^2^ SERVA Electrophoresis GmbH, Heidelberg, Germany

**Keywords:** glycan analytics, N-glycans, hofmann rearrangement, protein glycosylation, oligosaccharides, biopharmaceuticals

## Abstract

**Background:** Chemical methods for glycan release have gained traction because of their cost efficiency, accelerated reaction time and ability to release glycans not amenable to enzymatic cleavage. Oxidative chemical glycan release via hypochlorite treatment has been shown to be a convenient and efficient method that yields N-glycans similar to classical PNGase F digestion. We observed that the initial steps of the suggested mechanism for the oxidative release of glycans from glycoproteins by hypohalites showed similarities to the initiating steps of the classical Hofmann rearrangement of carboxamides. Therefore, we investigated the ability of different stable effectors of a Hofmann-type carboxamide rearrangement to efficiently and selectively release N-glycans from glycoproteins.

**Methods:** Released glycans obtained from different experimental chemical release approaches were analyzed by HILIC-FLD, BHZ-FACE and ESI-MS and evaluated with respect to electrophoretic mobility, retention time and integrated peak area for resolved glycans.

**Results:** We show that the known Hoffmann catalysts 1,3-dichloro-5,5-dimethylhydantoin, the hypervalent organoiodine (III) compound diacetoxy-iodobenzene as well as *in-situ* hypobromite generation using Oxone^®^ and potassium bromide are all capable of releasing protein-bound N-glycans in good yield. Among the compounds investigated, diacetoxy-iodobenzene was capable of releasing glycans in the absence of alkali. Detailed investigations of the bromide/Oxone^®^ method revealed a dependence of N-glycan release efficiency from the temporal order of bromide addition to the reaction mix as well as from a molar excess of bromide over Oxone^®^. Conclusions. These findings suggest that the oxidative release of N-glycans occurs via the initiating steps of a Hofmann carboxamide rearrangement. Hypervalent organoiodine compounds hold the promise of releasing glycans in the absence of alkali. The *in-situ* generation of hypobromite by bromide/Oxone^®^ produces a consistent defined amount of reagent for rapid N-glycan release for both analytical and preparative purposes.

## Introduction

Several methods have been described for the release of N-glycans from glycoproteins for functional analysis. While enzymatic approaches yield glycan samples of greater purity, the incurred costs of fast-acting enzymes are formidable for routine analysis. To overcome this issue, different chemical approaches for glycan release have been pursued. Most recently, oxidative release of intact glycans from glycoproteins and glycolipids by aqueous hypochlorite has been described (Song et al., 2016). Hypohalous acids and their salts, however, are rather unstable and difficult to be prepared, stored and handled in their solid anhydrous state due to a tendency to undergo exothermic disproportionation. As a result, the reagent content of a given aqueous hypochlorite preparation decreases over time as the molecule disproportionates into chloride ions and oxychlorine compounds with a higher oxidation number such as chlorite and chlorate (Lister, 1956). These instability issues of the reagent are undesirable for the application of hypohalite-mediated glycan release in validated assays where reproducibility and exact concentrations of reagents are critical. Therefore, we were interested in finding alternative chemical reagents that are commercially available in the solid state, have a long shelf life, are amenable to fine-tuned method development and method validation and yet capable of releasing N-glycans as efficiently as aqueous hypochlorite.

**FIGURE 1:**
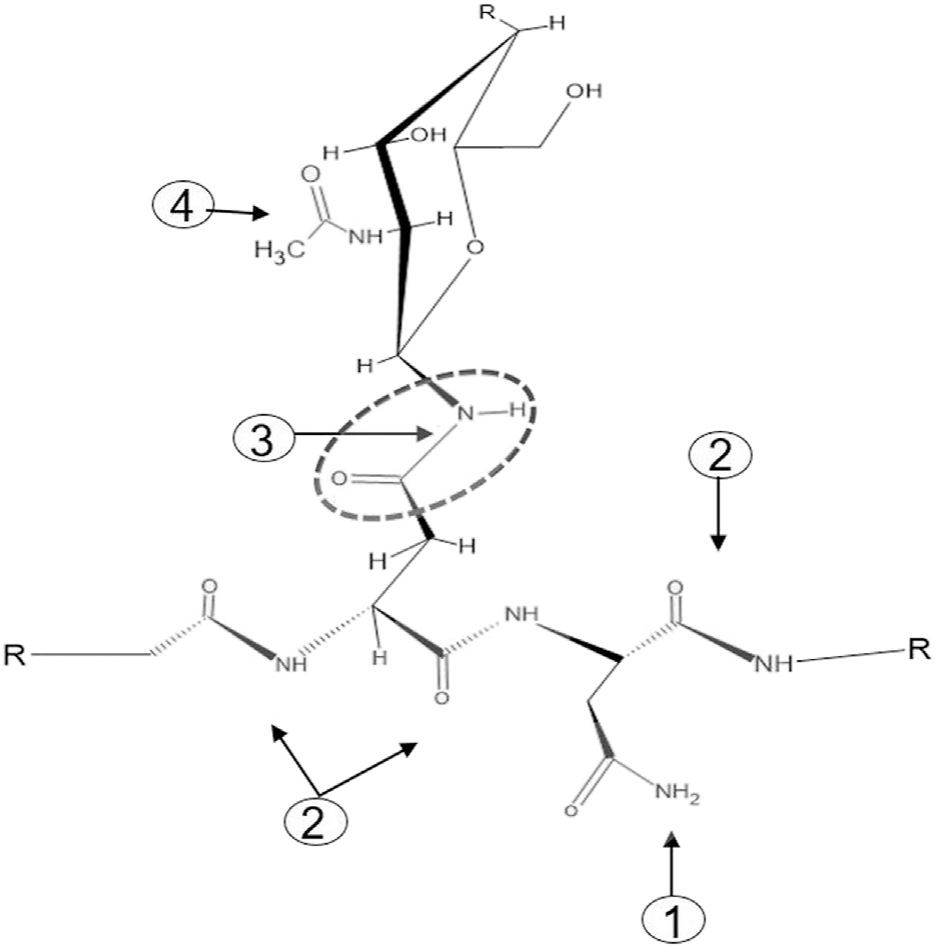
The four types of carboxamide structures in an N-glycoprotein. 1: Terminal free carboxamides of asparagine or glutamine side chains, 2: peptide bond carboxamides, 3: N-glycan-bonded asparagine-γ-carboxamide, 4 N-acetylglucosamine carboxamide.

One of the major challenges faced by chemical N-glycan release methods is the specificity of targeting the carboxamide linkage between the asparagine-γ-carboxamide and the bonded N-linked glycan. The numerous peptide bond carboxamides as well as the terminal free carboxamides of asparagine and glutamine side chains present in a glycoprotein add to the complexity of achieving chemical-based target specificity (Figure 1). Enzymatic removal of N-glycans with different N-glycanases overcomes these specificity issues as the catalytic function of N-glycanases—the hydrolysis of the carboxamide linkage between the asparagine-γ-carboxamide and the bonded core chitobiose N-glycan—is targeted to asparagine residues that reside in the glycosylation motif Asn-X-Ser/Thr. To date, two different types of N-glycanases or peptide-N-glycosidases (PNGases) are used routinely for N-glycan analysis. Both these PNGases, PNGase F and PNGase A, differ in their activity toward fucosylated N-glycans, with PNGase F recognizing only α-1,6-linked fusosylated core structures and PNGase A recognizing both α-1,6-linked and α-1,3-linked fusosylated structures (Altmann et al., 1998). The latter are found only in plant and invertebrate N-glycans making PNGase F the enzyme of choice for routine N-glycan analysis.

**FIGURE 2:**
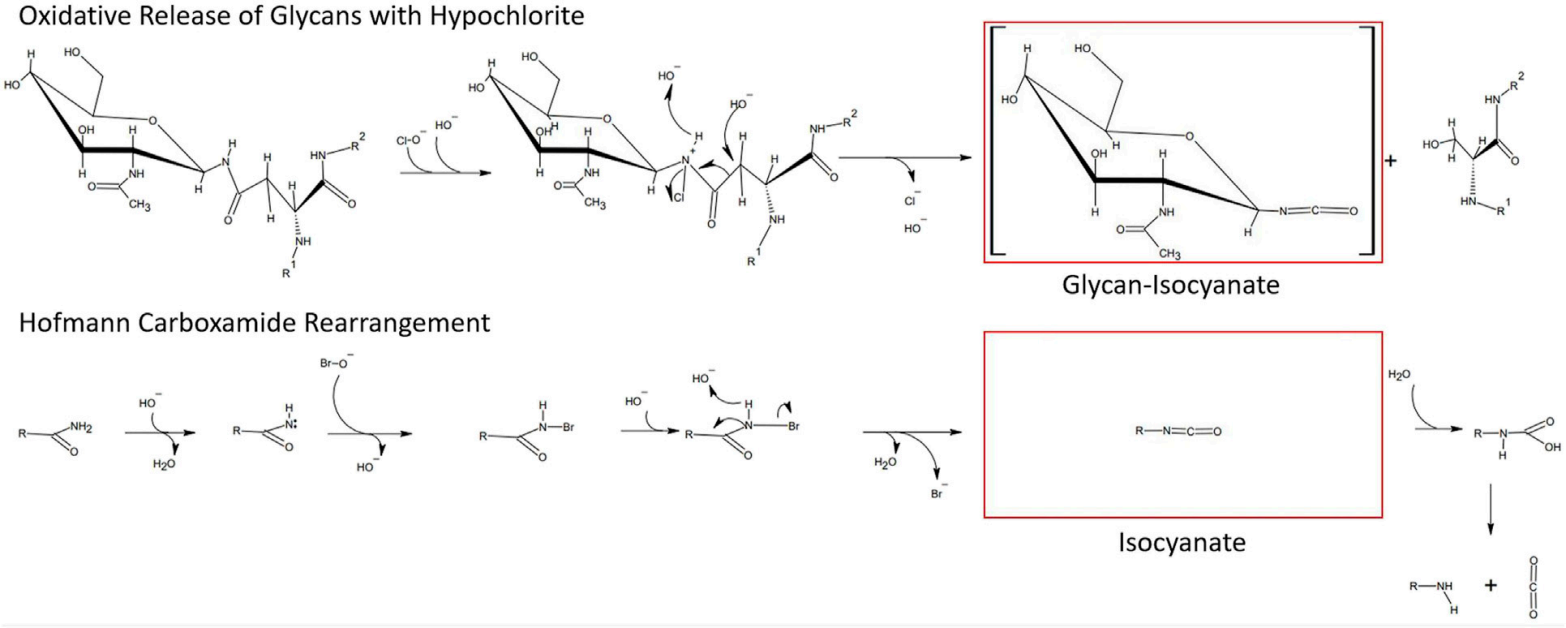
Similarities between the hypochlorite method and the Hofmann carboxamide rearrangement. (top) Proposed mechanism for hypochlorite mediated release of glycans. (bottom) Initiating steps in the Hofmann carboxamide rearrangement. Note that both methods proceed under highly alkaline conditions and *via* an isocyanate intermediate.

With the aim to identify chemicals capable of releasing N-glycans with a high degree of specificity toward the N-glycan bonded asparagine-γ-carboxamide, we focused on the proposed reaction mechanism for chemical release. Detailed analysis of the mechanism of oxidative glycan release proposed by Song et al. (2016) revealed similarities to the initiating steps of the classical Hofmann rearrangement of carboxamides (Hofmann, 1881). We observed that the N-chlorination by hypochlorite and the resultant generated isocyanate resembled the N-bromination and intermediate isocyanate formation during a classical Hofmann rearrangement of a primary caboxamide to a primary amine (Figure 2). In addition, both reactions proceed under highly alkaline conditions, with the pH of 1% aqueous hypochlorite being ∼13. Due to these similarities, especially in the initiating steps of the classical Hofmann rearrangement, we hypothesized that electrophilic chemical effectors of the Hofmann carboxamide rearrangement such as lead tetraacetate (Baumgarten et al., 1975), 1,3-dichloro-5,5-dimethylhydantoin (DCDMH) (Katuri and Nagarajan, 2019) and hypervalent organoiodine compounds such as *in situ* generated hydroxy(phenyl)iodonium ions (Zagulyaeva et al., 2010; Yoshimura et al., 2012), bis-trifluoroacetoxy iodobenzene (Radhakrishna et al., 1979; Almond et al., 1988) and diacetoxy-iodobenzene (Beckwith and Dyall, 1990; Zhang et al., 1997) would be effective in releasing N-glycans from glycoproteins. Thus, to obtain not only additional chemical tools for N-glycan release but also a better understanding of the mechanism behind, we tested different Hofmann reagents for their ability to efficiently and selectively break the bond between the γ-carboxamide-group of asparagine and the bound N-glycan. Surprisingly and in line with our proposed mechanistic analogy between oxidative glycan release and the Hofmann rearrangement, we found that different effectors of the Hofmann rearrangement cause a rapid and selective release of protein bound N-glycans. Here, we report on the different classes of chemical effectors of the Hofmann rearrangement that are capable of releasing N-glycans. We demonstrate the integrity of the released N-glycans by orthogonal analytical methods. Further, we describe a suitable chemical method for the efficient and selective release of N-glycans mediated by an optimized combination of potassium bromide and Oxone^®^. The chemicals investigated are commercially available in the solid state with an acceptable shelf life and thus can be specifically dosed to allow fine-tuned method development.

## Results

### 2 mg chicken ovalbumin releases sufficient N-glycans for robust fluorescence detection by RP-HPLC-FLD

**FIGURE 3:**
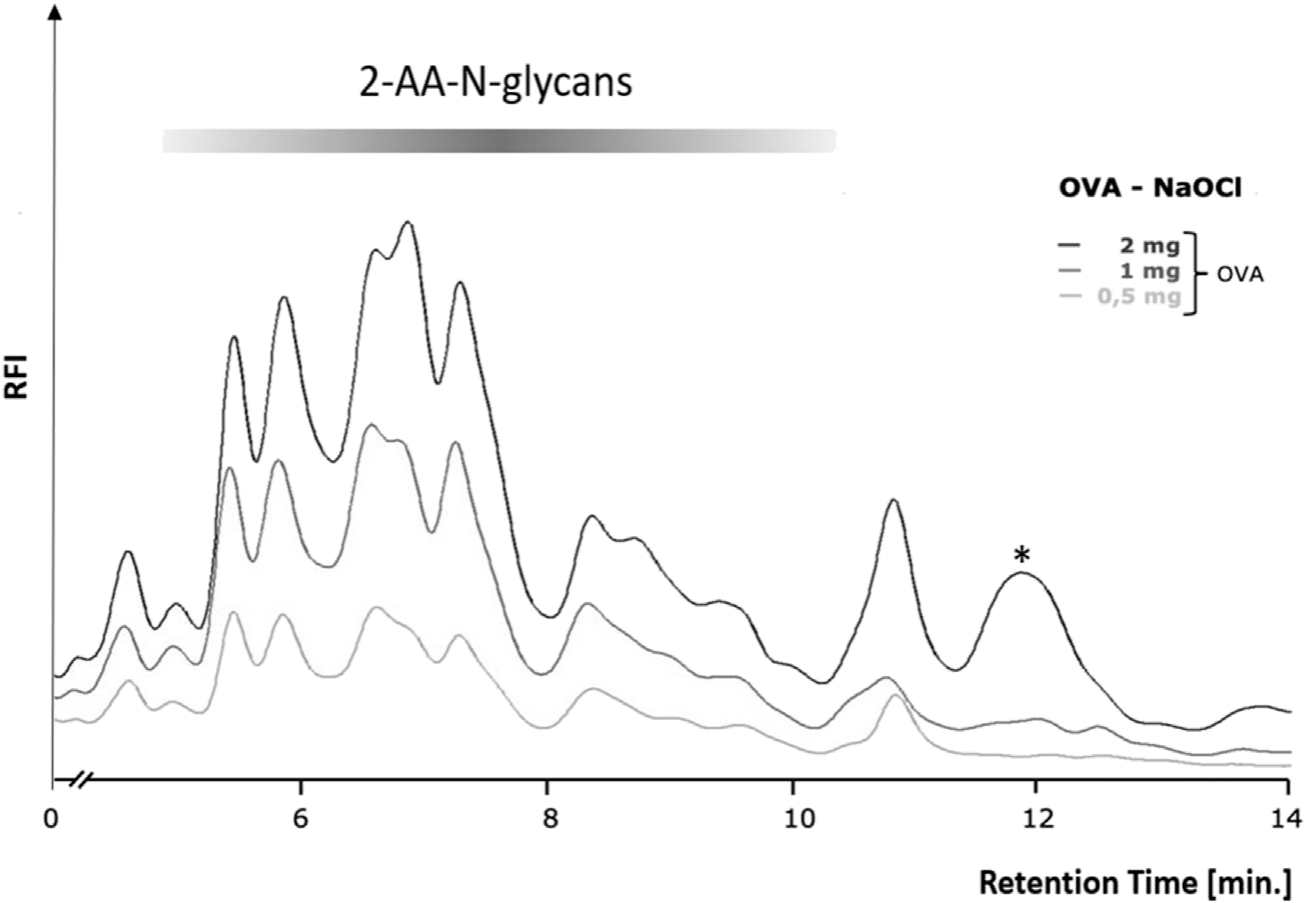
Glycoprotein concentration-dependent release of N-glycans by the hypochlorite method. Increasing amounts of chicken ovalbumin (OVA) from 0.5–2 mg yielded a similar RP-HPLC-FLD peak profile but with increasing relative fluorescence intensity (RFI) for the respective 2-AA-labeled N-glycans. At 2 mg ovalbumin, additional strong non-glycan artifact peaks (asterisks) emerged at retention times of 12 and 13.7 min. RP-HPLC-FLD analysis of 2-AA N-glycans was conducted under the following conditions: Column: OTU TriKala C18 150 × 3 mm, isocratic solvent: 10% ACN/0.1% formic acid, flow rate: 0.5 ml/min, column temperature: 25°C, fluorescence detection: *λ*_ex_ = 360 nm, *λ*_em_ = 420 nm; GAIN 100.

In order to first determine the optimum amount of a glycoprotein sample needed for robust and efficient glycan release by chemical methods, we conducted a glycoprotein concentration-dependent analysis with the established hypochlorite method published by Song et al. (2016). We chose to use chicken ovalbumin for these studies as this protein is inexpensive, readily available in large amounts, and the N-glycans have been well characterized. We first tested different reaction times and temperatures and found a reaction time of 10 min and an incubation temperature of 37°C to yield the best results (Supplementary Figure S1). Varying amounts of chicken ovalbumin were then incubated with hypochlorite to release the N-glycans. The released N-glycans were purified by Carbograph-SPE and then labeled with 2-aminobenzoic acid (2-AA) by reductive amination. The labeled N-glycans were further purified to remove excess label and then analyzed by C18-RP-HPLC-FLD. Figure 3 shows that increasing amounts of chicken ovalbumin glycoprotein yield linearly increasing amounts of 2-AA-labeled N-glycans detectable by C18-RP-HPLC-FLD. The highest relative fluorescence intensity per normalized glycan sample was observed for 2 mg ovalbumin per assay, although a significant increase in peak area for a non-glycan artifact (asterisk, Figure 3) could also be observed. Based on these findings we decided not to further increase the glycoprotein amount and to conduct all experiments with a maximum of 2 mg glycoprotein per assay.

### Different chemical effectors of the Hofmann carboxamide rearrangement release protein-bound N-glycans from chicken ovalbumin

**FIGURE 4:**
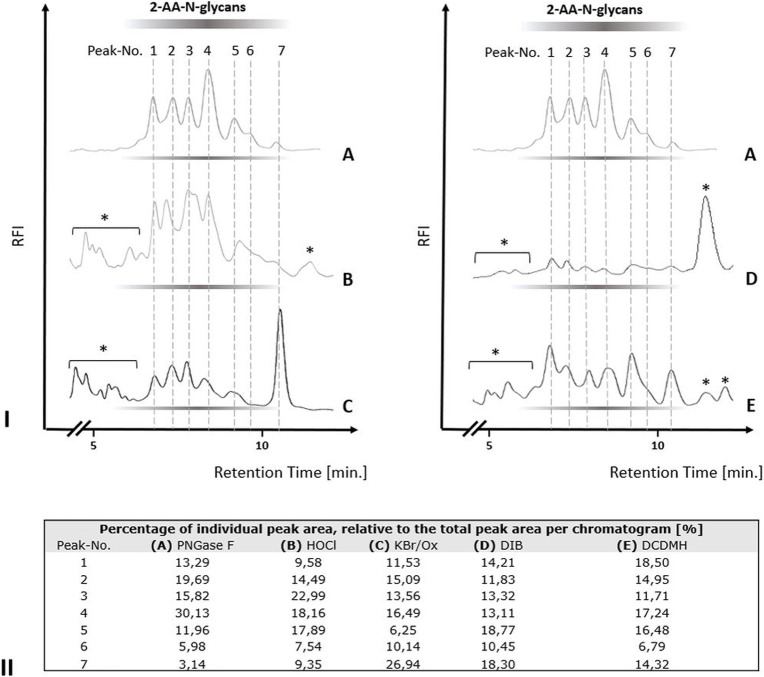
N-glycan release from chicken ovalbumin caused by different chemical effectors of the Hofmann carboxamide rearrangement. I. RP-HPLC-FLD peak profiles for the respective 2-AA-labeled N-glycan samples obtained by enzymatic or by the different chemical methods: **(A)**: Positive control, PNGase F released 2-AA- N-glycans, **(B)**: Hypochlorite released 2-AA-N-glycans, **(C)**: Bromide/Oxone released 2-AA-N-glycans, **(D)**: Diacetoxy-iodobenzene (DIB) released 2-AA-N-glycans, **(E)**: 1,3-dichloro-5,5-dimehylhydantoin (DCDMH) released 2-AA-N-glycans. Asterisks: non-glycan artifacts. RP-HPLC-FLD conditions: column: OTU TriKala C18 150 × 3 mm, isocratic solvent: 10% Acetonitrile/0.1% formic acid, flow rate: 0.5 ml/min., column temperature: 25°C, *λ*_ex_ = 360 nm, *λ*_em_ = 420 nm; GAIN 100. II. Percentages of individual peak area for each chromatogram of Figure 4I, relative to the total peak area per each chromatogram [%].

We next tested the three different classes of known Hofmann-reagents—1,3-dichloro-5,5-dimethylhydantoin (DCDMH), Oxone^®^ and potassium bromide (Ox/KBr), and diacetoxy-iodobenzene (DIB)—for their ability to break the bond between the asparagine-γ-carboxamide and the N-linked glycan. We incubated 2 mg ovalbumin with either PNGase F, hypochlorite, DCDMH, Ox/KBr or DIB. The N-glycans released by these different methods were then labeled with 2-AA via reductive amination, purified from excess label and resolved by C18-RP-HPLC-FLD (Figure 4). Figure 4 shows the resolved N-glycan peaks obtained by the two benchmark methods—N-glycans released enzymatically by PNGase F and chemically by hypochlorite—and the RP-HPLC-FLD peak profiles obtained by the different chemical effectors of the Hofmann rearrangement. Figure 4IA shows the RP-HPLC-FLD peak profile for 2-AA labeled N-glycans released by PNGase F from chicken ovalbumin. In RP-HPLC-FLD, the 2-AA labelled glycans elute in the order of decreasing hydrophilicity, i.e., the more complex and larger glycan oligosaccharides elute first while the smaller glycan oligosaccharides elute later. This means that the early eluting peaks represent larger glycan oligosaccharide species while the smaller glycan structures elute late. It must be noted that these peaks do not necessarily reflect individually resolved glycan species but rather a mix of glycan species that elute at a similar retention time and yet in an order of decreasing hydrophilicity. The scouting RP-HPLC profile for PNGase F released ovalbumin N-glycans resolves into a pattern of seven peaks which were numbered in the order of their elution from the column.

As expected, the retention times of the ovalbumin N-glycan peaks from the PNGase F treated sample (Figure 4IA) were in alignment with the retention times of the peaks obtained by hypochlorite treatment (Figure 4IB). Remarkably, all of the other three effectors of a Hofmann rearrangement that we tested—DCDMH, DIB and Ox/KBr—displayed N-glycan peak profiles that matched the retention times of the glycan peaks obtained by both PNGase F and hypochlorite. Interestingly however, the peak intensities for the released ovalbumin N-glycans vary among the different methods (Figures 4I,II). Figure 4II shows the percentage of each individual peak area for the numbered peaks in relation to the total combined peak area of each respective chromatogram. The large glycan species of peak one had the highest relative percentage relative to the total spectrum of released glycans in the sample released by DCDMH (Figure 4II). The RP-HPLC-FLD pattern of 2-AA labelled N-glycan peaks released from chicken ovalbumin is resembling a lower resolution of previously published results for chicken ovalbumin 2-AB glycans resolved by RP-HPLC (Chen and Flynn, 2009). The major peak four observed for the PNGase F released ovalbumin N-glycans was much less prominent in the samples that were released by hypochlorite (Figure 4IB), Oxone/bromide (Figure 4IC), DIB (Figure 4ID) and DCDMH (Figure 4IE). Also the late eluting peak 7 appeared to be most dominant in the Oxone/bromide sample (Figures 4IC,II). We observed that the N-glycan peaks released by DIB (Figure 4ID) showed considerably diminished relative fluorescence intensity compared to DCDMH (Figure 4IE) and Ox/KBr (Figure 4IC). Here, it must be noted that while the reaction conditions and times with hypochlorite, DCDMH and Ox/KBr were identical with pH > 10 and a reaction time of 10 min, the N-glycans released with DIB were obtained upon prolonged incubation (72 h) at pH 7.5. At shorter incubation times no N-glycan peaks were detected.

### A substantial molar excess of bromide over oxone^®^ increases the efficiency of N-glycan release

**FIGURE 5:**
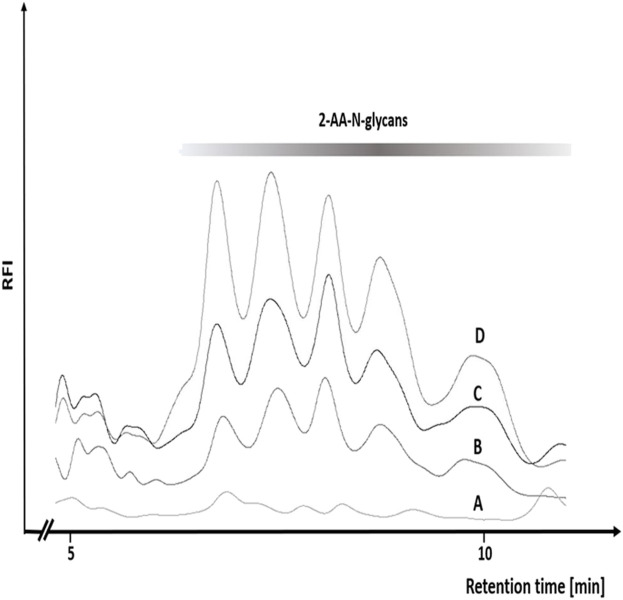
Increasing concentrations of bromide result in a more efficient N-glycan release from chicken ovalbumin by the Oxone^®^/Bromide method. RP-HPLC-FLD peak profiles for the respective 2-AA-labeled N-glycans released by either Oxone^®^ alone **(A)**, Oxone^®^/Bromide mixture with 2.1 **(B)** 3.3 **(C)** and 14.4 **(D)** molar excess of bromide: Oxone^®^. Column: OTU TriKala C18 150 × 3 mm, isocratic solvent: 10% Acetonitrile/0.1% formic acid, flow rate: 0.5 ml/min., column temperature: 25°C, fluorescence detection: *λ*_ex_ = 360 nm, *λ*_em_ = 420 nm; GAIN 100.

In our initial experiments with the Ox/KBr system we observed a rather surprising dependency of the efficiency of N-glycan release on the sequential order of reagent addition. Specifically, optimal N-glycan release occurred only when bromide was first premixed with the glycoprotein prior to addition of Oxone^®^. A premix of Oxone^®^ and bromide added to the glycoprotein consistently yielded very low amounts of N-glycans (data not shown). This finding was initially surprising as we had considered hypobromite to be the active reagent effecting the Hofmann-mediated N-glycan release. However, this points to the instability of a hypobromite solution and its need to be generated *in situ* in the presence of glycoprotein and directly at the site where it is required. This further suggested that the hypobromite concentration may influence the efficiency of N-glycan release. Based on this, we tested whether increasing the concentration of bromide could result in a more efficient N-glycan release. Our data show that Oxone^®^ alone—at an equimolar concentration to 0.5% (w/v) hypochlorite—released N-glycans at very low levels (Figure 5, Chromatogram A). Increasing the ratio of bromide:Oxone^®^ from 2.1-fold to 14.4-fold molar excess dramatically increased the efficiency of glycan release from ovalbumin (Figure 5, Chromatograms B-D).

### Orthogonal methods confirm the integrity of the N-glycans released by the oxone^®^/bromide method

**FIGURE 6:**
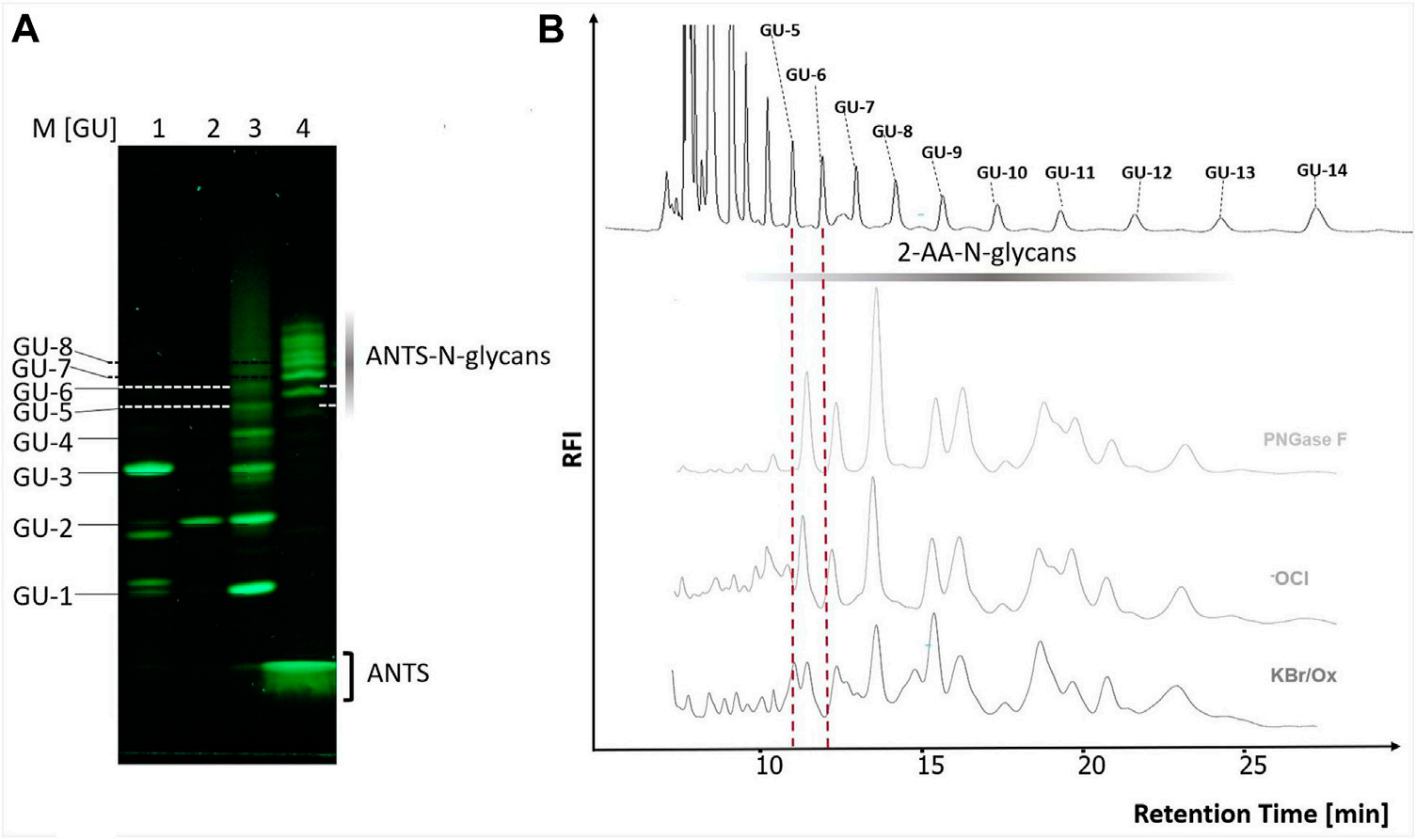
The chicken ovalbumin N-glycan profiles released by PNGase F and Oxone^®^/Bromide are identical by FACE and HILIC-FLD analysis. **(A)** BHZ-FACE Electropherogram showing the separation of ANTS-labeled mono-, di- and trisaccharides, dextran-hydrolysate marker and PNGase F released N-glycans from chicken ovalbumin. Lane 1: Resolved mix of ANTS-labeled mannose, lactose and maltotriose, Lane 2: ANTS-labeled maltose Lane 3: ANTS-labeled dextran partial hydrolysate standard, Lane 4–6: ANTS-N-glycans released from chicken ovalbumin by PNGase F, hypochlorite and Ox/KBr, respectively. *λ*_ex_ = 365 nm, recorded by 12Mpx CMOS camera. **(B)** HILIC-FLD peak profiles for 2-AA-labeled N-glycans released by either PNGase F, hypochlorite or Oxone^®^/Bromide mixture with a 3.3 molar excess of bromide: Oxone^®^. Column: OTU Amino 250 × 4.6 mm, isocratic solvent: 60 % ACN / 10 mM ammonium formate (pH 4.4) flow rate: 0.5 ml/min., column temperature: 25°C, fluorescence detection: *λ*_ex_ = 360 nm, *λ*_em_ = 420 nm; GAIN 100.

To further confirm the integrity of the N-glycans released by the optimized Oxone^®^/Bromide method by orthogonal methods, we tested the following: 1. Blue Horizon Fluorescence Assisted Carbohydrate Electrophoresis (BHZ-FACE) of 8-aminonaphthalene-1,3,6-trisulfonate (ANTS)-labeled N-glycans and 2. HILIC-FLD analysis of 2-AA-labeled N-glycans. ANTS- and 2-AA labeled partial dextran hydrolysate served as a calibration standard for the electrophoretic mobility of ANTS-labeled glucose units (GU-1 to GU-8) and the retention time of 2-AA labeled glucose units (GU-5 to GU-14) on HILIC-FLD (Figure 6), respectively. In line with previously published reports on the heterogenic N-glycan composition of chicken ovalbumin, PNGase F released ovalbumin N-glycans detected by BHZ-FACE and HILIC-FLD started to resolve between GU-5 and GU-6 and ended by GU-13 (Figures 6A,B) (Chen and Flynn, 2009). The slight deviation of N-glycan bands and peaks from the GU-standard bands matching the identical hexose unit amount is a known phenomenon that could either be attributed to the presence of desoxyhexoses or to the branched nature of the natural N-glycans (Guile et al., 1996; Harvey et al., 2000). (Figure 6A,B) show the overlap in band patterns and peak profiles of N-glycans released from chicken ovalbumin by either PNGAse F, hypochlorite or Oxone^®^/Bromide.

### The oxone^®^/bromide system releases Fc-N-glycans from an IgG1-isotype monoclonal antibody

**FIGURE 7:**
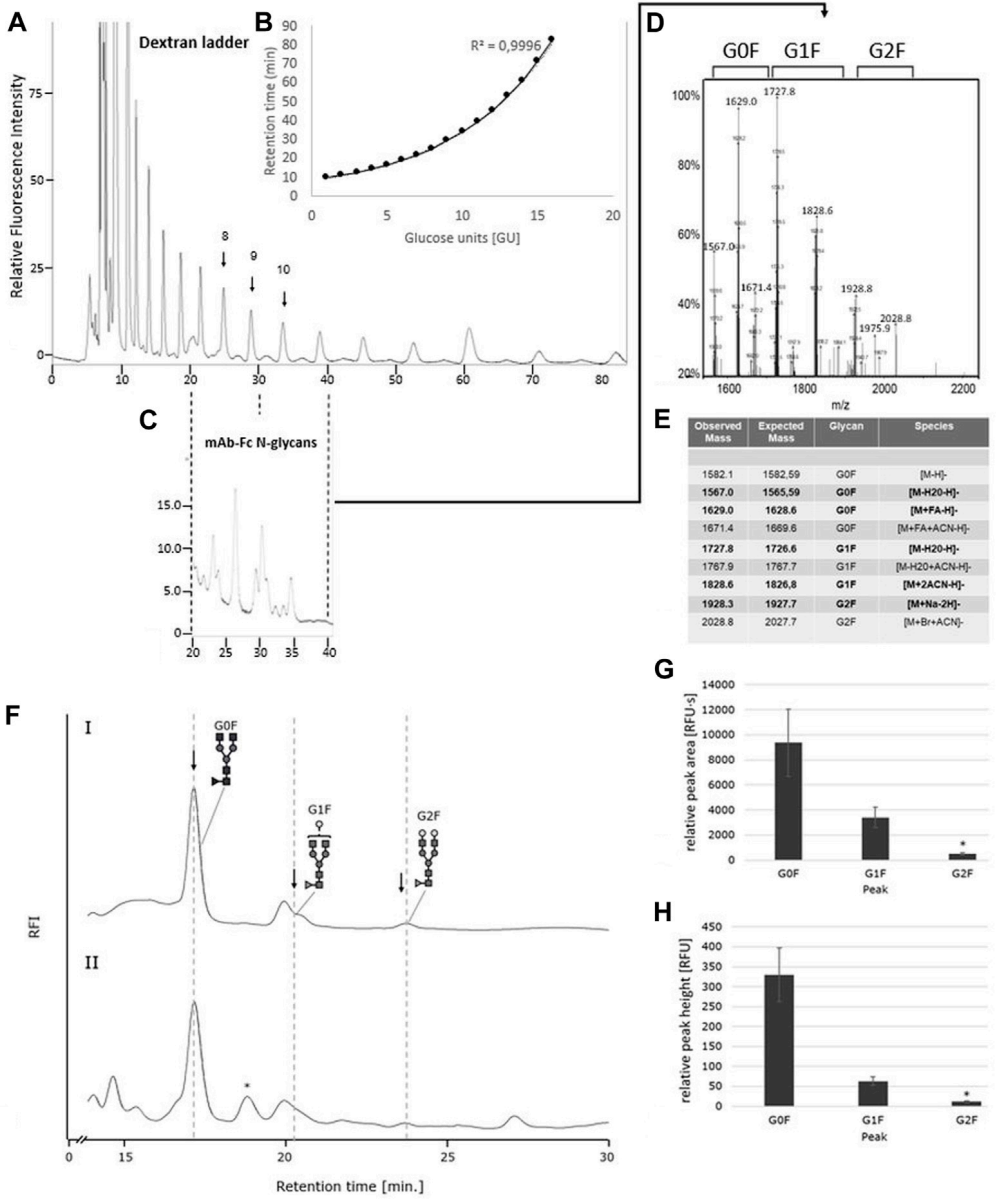
The IgG1-mAb-Fc N-glycan profiles released by PNGase F and Oxone^®^/Bromide are identical by HILIC-FLD analysis. **(A)** HILIC-FLD Separation of 2-AA labelled dextran standard at a flow rate of 0.5 ml with indication of individual retention times for hexose unit peaks (GU units). **(B)** Plot of HILIC retention times versus size of hexose units. Note the direct correlation between HILIC retention time and size of the linear oligosaccharides. **(C)** HILIC-FLD chromatogram of 2-AA glycans derived from IgG1-Fc. Peaks for G0F, G1F and G2F elute in the vicinity of the respective GU unit standard peak. **(D)** ESI-MS spectrum for the collected HILIC peaks for the IgG1-Fc glycans. **(E)** ESI-MS peak assignment for the ESI-MS peaks shown in panel **(D,F)**. HILIC-FLD peak profiles for 2-AA-labeled N-glycans released by PNGase F (chromatogram FI) or Oxone^®^/Bromide (chromatogram FII). Column: OTU Amino 250 × 4.6 mm, isocratic solvent: 60 % ACN / 10 mM ammonium formate (pH 4.4), flow rate: 0.5 ml/min (Panel **A,C**). 1 ml/min (Panel **F**), column temperature: 25°C, fluorescence detection: *λ*_ex_ = 360 nm, *λ*_em_ = 420 nm; GAIN 100. Bottom panels: Mean values and standard deviation for peak area **(G)** in relative fluorescence units x second [RFU·s] and peak height **(H)** in relative fluorescence units [RFU] for the individual peaks (*n* = 3). * *n* = 2.

Having established the Oxone^®^/Bromide method for chicken ovalbumin, we next investigated the ability of this method to release N-glycans from another glycoprotein, namely an IgG1-type monoclonal antibody (mAb). PNGase F digestion of the mAb yielded the well-known pattern of a predominant G0F biantennary glycan, the two G1F structural variants and a minor G2F-glycan structure (Figures 7C,FI). The identity of the glycan species was confirmed by HILIC FLD retention time and ESI-MS (Figures 7A–E). Interestingly, mAb-Fc N-glycans released by the Oxone^®^/Bromide method yielded a largely similar pattern (Figure 7FII). The glycan peaks for G0F and G1F were easily detectable and matched those obtained with PNGase F. However, the G2F peak in the Oxone^®^/Bromide sample was barely detectable and two additional peaks appeared at 19 and 27 min retention time that were not present in the PNGase F treated mAb-Fc-N.glycan sample. The consistency and robustness of the Oxone^®^/Bromide method is exemplified in the mean peak area and height values for G0F, G1F and G2F from three independent treatments as shown in Figures 7G,H.

### The oxone^®^/bromide system releases homogeneous core-F paucimannose-type N-glycans more efficiently compared to heterogeneous N-glycans

**FIGURE 8:**
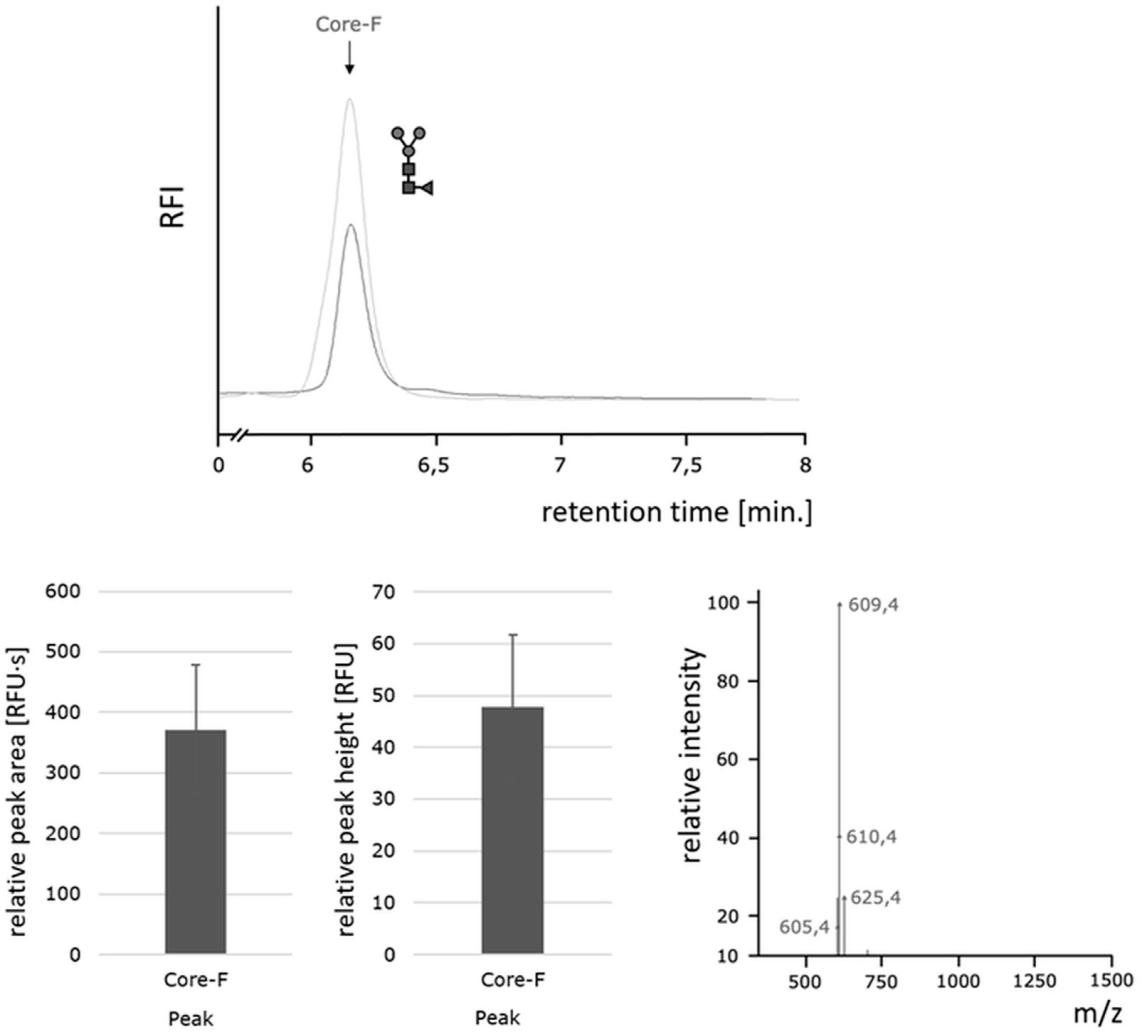
Homogeneous paucimannose core-F N-glycans released by the Oxone^®^/Bromide method. Top: HILIC-FLD peak profiles for 2-AA-labeled N-glycans released from RSVF by either PNGase F (light grey trace) or Oxone^®^/Bromide (dark grey trace). Column: OTU Amino 250 × 4.6 mm, isocratic solvent: 60 % ACN / 10 mM ammonium formate (pH 4.4), flow rate: 0.5 ml/min., column temperature: 40°C, fluorescence detection: *λ*_ex_ = 360 nm, *λ*_em_ = 420 nm; GAIN 100. Bottom left: Mean values and standard deviation for peak area (left) in relative fluorescence units x second [RFU·s] and peak height (right) in relative fluorescence units [RFU] for the core F peak (with *n* = 3). Bottom right: Positive ion mode m/z data for the 2-AA labeled core-F glycan.

To further investigate the efficiency of the Oxone^®^/Bromide method toward releasing different N-glycans, we used the BEVS/Sf-9-derived ectodomain of the Respiratory Syncytial Virus F-protein (RSVF), a glycoprotein containing three N-glycosylation sequons, each occupied by a homogeneous core-F-type paucimannose glycan structure (Sandig et al., 2015). Thus, the N-glycan profile of this glycoprotein shows a single peak and is in stark contrast to the heterogeneous profile of chicken ovalbumin. Our data show that PNGase F as well as the Oxone^®^/Bromide method both released an identical single predominant N-glycan structure (Figure 8, top panel). The consistency and robustness of the Oxone^®^/Bromide method is again evident in the mean peak area and height values from three independent treatments as shown in Figure 8 (bottom left panel). The identity of the released glycan as a Core-F-type paucimannose structure was confirmed by ion trap MS (Figure 8, bottom right panel). The obtained mass-to-charge ratio of 609.4 m/z corresponds to a doubly protonated adduct of the 2-AA-labeled core-F N-glycan structure and an acetonitrile solvent molecule[M + ACN+2H] (expected m/z ratio = 609.8 m/z).

**FIGURE 9:**
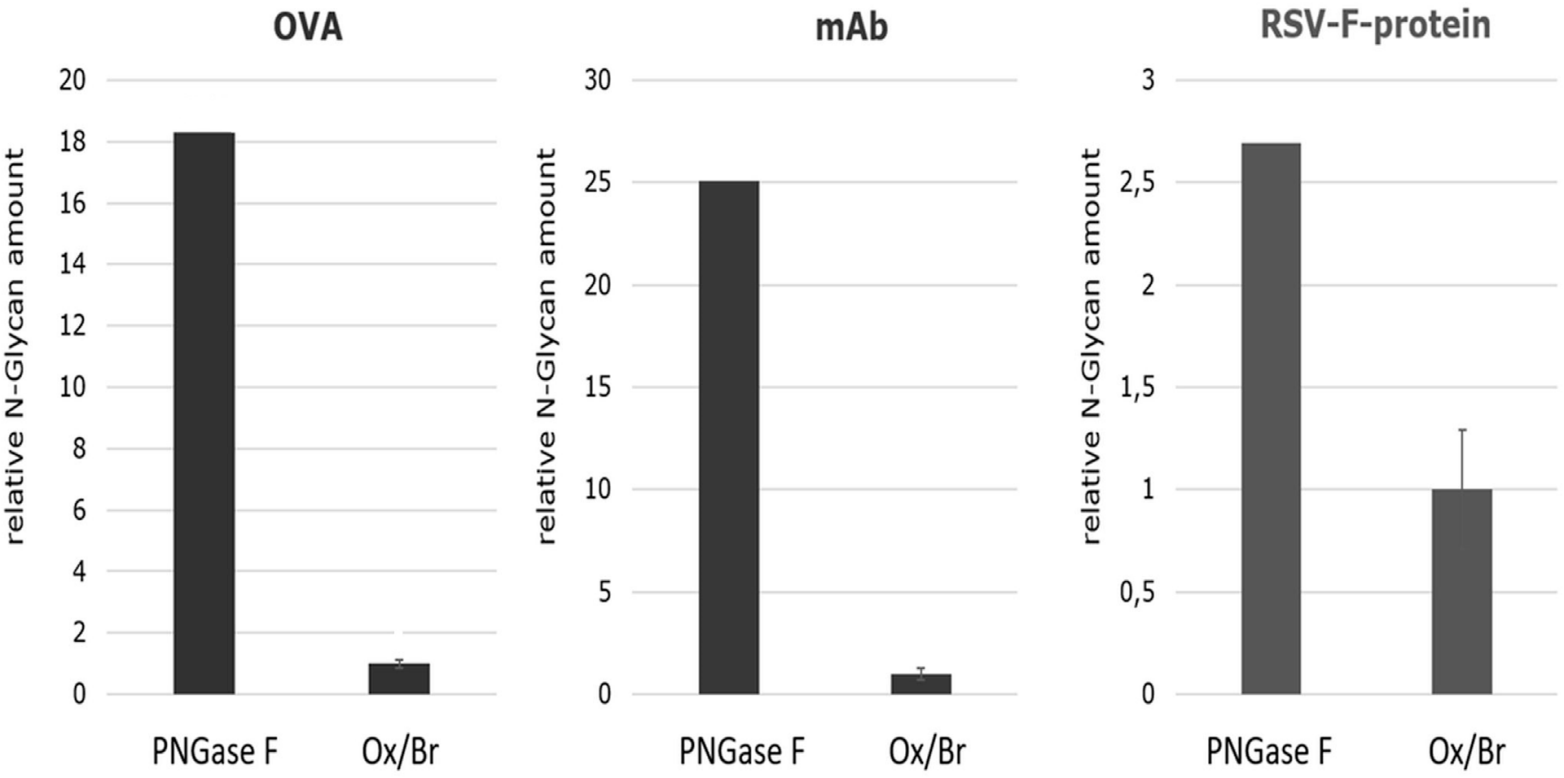
Comparison of the N-glycan release efficiency between the Oxone^®^/Bromide and PNGase F method for three different glycoproteins based on the relative N-glycan amount. The cumulative peak area of all N-glycan peaks obtained by either the Oxone^®^/Bromide or by PNGase F treatment was obtained for each of the three different glycoproteins—chicken ovalbumin, IgG1-monoclonal antibody and RSVF. The relative N-glycan amount was determined by the sum of the peak area of the major N-glycan form of the respective glycoproteins. For the KBr/Ox method, the mean value including standard deviation (*n* = 3) was determined, the value normalized to 1, and plotted relative to PNGase F. Shown on the left is the relative N-glycan amount of ovalbumin (OVA) (sum of peak area of 10 glycan peaks), in the middle of IgG1-monoclonal antibody (mAb) (sum of peak area of G0F, G1F, G2F) and on the right of RSVF protein (peak area of Core-F) of the two release methods. -

In order to estimate the efficiency of the Oxone^®^/Bromide method in comparison to PNGase F, the cumulative N-glycan peak area for each of the three different glycoproteins—chicken ovalbumin, IgG1-monoclonal antibody and RSVF—from both these treatments were obtained and the mean values from three independent experiments calculated. We assumed that the cumulative peak area represents the total amount of N-glycans released and thus is indicative of the overall efficiency of the method. These values were then normalized to the amount of N-glycans released from chicken ovalbumin by the Oxone^®^/Bromide method that was set to 1.0. Figure 9 shows that the Oxone^®^/Bromide method releases approximately 18-fold less N-glycan structures from chicken ovalbumin and 25-fold less N-glycan structures from IgG1-mAb compared to PNGase F (Figure 9, left and middle panel, respectively). In contrast to complex heterogeneous N-glycan structures, the Oxone^®^/Bromide mediated N-glycan release from the homogeneously glycosylated RSVF protein was only 2.5-fold less efficient compared to PNGase F (Figure 9, right panel).

## Discussion

The selective release of protein-bound glycans by chemical means is highly desirable because of digestion speed and cost benefits. In order for these chemicals to be used in validated analytical methods in the biopharmaceutical industry, these reagents must be stable, have a long shelf-life, be suitable for fine-tuned method development, be effective and more importantly selective in releasing glycans from their covalently linked protein structures. We focused our efforts on three different classes of reagents capable of effecting a Hofmann rearrangement. The first group of reagents were N-chloro compounds known to hydrolyze in solution to yield hypochlorite. These also fit our requirements for being a stable, solid, easily dosable source of hypohalites. While the most well-known of these compounds, tosylsulfonylazanide (Chloramine T) yields just 1 mol of hypochlorite per mole chloramine T with rather slow reaction kinetics, the oxidizing heterocyclic agents 1,3-dichloro-5,5-dimethylhydantoin (DCDMH) and trichloroisocyanuric acid (TCCA) rapidly hydrolyze to yield a two- and three times molar excess of hypochlorite per mole DCDMH and TCCA, respectively. DCDMH had also been successfully employed as a key reactant in a Hoffmann rearrangement reaction (Katuri and Nagarajan, 2019). In the second group was the strong oxidizing agent Oxone^®^ (Pentapotassium bis(peroxymonosulphate) bis(sulphate); 2KHSO_5_•KHSO_4_•K_2_SO_4_) that was known to be capable of quantitatively releasing the halogens iodine, bromine and chlorine from their respective halides. This reaction proceeds under alkaline conditions and the hypohalites–the classical Hofmann reactants-are formed *in situ*. Finally, in the third class of reagents were the hypervalent organoiodine compounds that have also long been known to be effective in inducing a Hofmann-type carboxamide rearrangement (Zhang et al., 1997; Zagulyaeva et al., 2010; Yoshimura et al., 2012). This group held the promise of effecting the release of N-glycans under mild non alkaline conditions. Bis(trifluoroacetoxy)iodobenzene was the first of these compounds to be used for converting amides into amines under mild neutral to slightly acidic conditions (Radhakrishna et al., 1979; Almond et al., 1988). Later, diacetoxy-iodobenzene (DIB) was described as a mild and selective reagent for effecting the Hofmann rearrangement of carboxamides at room temperature without attacking any other oxidizable functional groups (Beckwith and Dyall, 1990). Interestingly, this selectivity feature of DIB was capitalized to develop a method for the DIB-mediated selective cleavage of peptide bonds preceding an asparagine residue in neutral aqueous solution at 37°C (Tanabe et al., 2014).

Thus, we investigated the ability of 1,3-dichloro-5,5-dimethylhydantoin (DCDMH), Oxone/bromide mixtures, and the hypervalent organoiodine (III) compound diacetoxy-iodobenzene (DIB) to release glycans from glycoproteins. We provide evidence for chemical effectors of the Hofmann rearrangement in effecting glycan release and report on an optimized method using Oxone^®^ and potassium bromide that efficiently releases glycans from different glycoproteins. The suitability of these chemicals to method development points toward the applicability of this method for biopharmaceutical purposes.

In the classical Hofmann rearrangement reaction, the amide group first reacts with the halogen reagent to form an N-halogenated amide with an electron-deficient nitrogen atom, which in turn is deprotonated under alkaline conditions, undergoes further rearrangement to form an isocyanate intermediate species (Figure 2). Based on the proposed mechanism for the cleavage of N-glycan-γ-carboxamide bonds by hypochlorite, we noted its similarity to the initial steps of the Hofmann rearrangement mechanism, in particular the alkaline conditions and the formation of an isocyanate intermediate (Song et al., 2016). Both in the Hofmann rearrangement as well as the oxidative release of protein bound N-glycans, hypohalites are the key active reagents in triggering the carboxamide conversion. Indeed, in the first description of the Hofmann reaction by August Wilhelm von Hoffmann in 1881, *in situ* generated hypobromite was originally used (Hoffmann, 1881). Interestingly, the rate of rearrangement in a Hofmann reaction was found to be directly proportional to the reaction temperature, the concentration of alkali present and the electronegativity of the leaving group halogen, which in turn determines the rate limiting step—the release of the halogen from the N-haloamide intermediate product (Renfrow and Hauser, 1937; Marzluff, 1947). The latter point suggests that hypochlorite should be a more effective reagent due to the chlorine atom being a better leaving group making the N-chloroamide intermediate more unstable than the cognate N-bromoamide, (Marzluff, 1947). Hypochlorite treatment resulted in complete degradation of the glycoprotein, indicative of an aggressive reactivity toward all three types of carboxamides present in a glycoprotein (Figure 1) (Song et al., 2016). We hypothesized that the electronegativity of the leaving group halide and the efficiency of its release from the N-haloamide intermediate product could also potentially impact the selectivity of the N-glycan cleavage reaction. After all this N-haloamide degradation is considered to be the rate limiting step in the Hofmann carboxamide rearrangement (Renfrow and Hauser, 1937; Marzluff, 1947). The terminal carboxamides of asparagine and glutamine side chains are not cleavable and therefore are irrelevant for our purposes. The carboxamide linkage between the N-glycan and the asparagine-γ-carboxamide, however, is unique among the cleavable carboxamide linkages in a glycoprotein as the lone electron pair at this carboxamide nitrogen atom is subject to a much stronger -I-effect that originates from the multiple oxygen atoms in the adjacent carbohydrate moiety. In turn this means that this particular nitrogen atom is less likely to let go of its electrons thereby limiting the successful cleavage rate for this carboxamide bond. Peptide bond carboxamides in turn are more likely to get cleaved. The more electronegative the halide atom of the key reagent, the more extensive the inadvertent cleavage of peptide bond carboxamides. We therefore expected hypobromite and hypoiodite to be less aggressive and possibly more selective reagents in releasing N-glycans via Hofmann-type carboxamide rearrangement. However, *in-situ* generated hypoiodite from equimolar amounts of potassium iodide and potassium peroxodisulfate under alkaline conditions was ineffective in releasing protein bound glycans (data not shown). This could be a direct consequence of the high pKa of the hypoiodus acid. On the other hand, under alkaline conditions, *in-situ* generated hypobromite was highly effective in releasing protein bound glycans. Hypobromous acid has a pKa value (pKa 8.7) intermediate between hypoiodous and hypochlorous acid. Hypobromite is also known for its stronger bleaching performance in high pH as compared to hypochlorite (Jewson, 1954; Dallmier, 1997). However, unlike sodium hypochlorite which remains stable in solution at room temperature over short periods, sodium hypobromite is highly unstable under typical storage conditions and quantitatively disproportionates into bromate and bromide at temperatures above 20°C (Marzluff, 1947; Jewson, 1954; Dallmier, 1997). Stable solutions of hypobromite can only be maintained in the presence of alkali sulfamates (Dallmier, 1997). Therefore, to overcome this issue, we chose to generate hypobromite *in situ* from Oxone^®^ and potassium bromide under alkaline conditions. As we could demonstrate (see Figures 5–7), this procedure is highly effective and robust in providing the required amounts of hypohalite species to achieve cleavage of the N-glycan carboxamide. Due to the spontaneous disproportionation of aqueous hypobromite into bromide and bromate at temperatures above 20°C, all reagents were maintained on ice prior to mixing. It was further necessary to premix potassium bromide with the glycoprotein prior to Oxone^®^ addition. With this order of reagent addition, hypobromite could be generated *in situ* in the presence of glycoprotein, and loss of hypobromite due to disproportionation minimized. We also discovered that a molar excess of bromide over Oxone^®^ greatly increased the efficiency of glycan release (Figure 5). This could be explained by a reversal of hypobromite disproportionation caused by elevated bromide via Le Chatelier’s principle. Hypervalent iodine species have also been employed in the Hoffman reaction. Therefore, we tested diacetoxy-iodobenzene (DIB), a classic hypervalent organoiodine compound in its ability to selectively break the bond between the γ-carboxamide-group of asparagine and the bound N-glycan under varying reaction conditions. DIB is a well-known effector of the Hofmann carboxamide rearrangement and capable of forming N-iodine (III) species with carboxamide groups (Zhang et al., 1997; Tanabe et al., 2014). Our data show that DIB is capable of breaking the bond between the asparagine-γ-carboxamide and the bonded N-glycan moiety (Figure 4ID). Unfortunately, the observed reaction kinetics were rather slow and required a reaction time of 72 h. Fluorinated hypervalent organoiodine compounds such as bis(trifluoroacetoxy)iodobenzene or the addition of fluorinated catalysts may have the potential to accelerate the reaction kinetics of hypervalent organoiodine mediated glycan release. Other hypervalent iodine species that can be generated *in situ* from low cost reactants also hold potential. The *in-situ* production of hydroxyphenyliodonium-ions and its immediate use as a catalyst in a carboxamide rearrangement reaction was recently described (Zagulyaeva et al., 2010; Yoshimura et al., 2012). This cost-efficient method for inducing a selective Hofmann rearrangement with hypervalent iodine species works by mixing stoichiometric amounts of iodobenzene and Oxone^®^ to yield [(hydroxy(phenyl)iodonium ion) PhI(OH]+ as a reactive species *in situ* at room temperature in aqueous acetonitrile and 1,1,1,3,3,3-hexafluoroisopropanol (Zagulyaeva et al., 2010; Yoshimura et al., 2012). The highly fluorinated alcohol had a catalytic accelerating effect on the hypervalent iodine mediated conversion of carboxamide to amine (Middleton, 2013). With regard to chemical glycan release methods, hypervalent iodine reactants hold the promise of acting under neutral to acidic conditions and thereby allowing for the release of intact glycans that are not subject to alkaline peeling, a base-catalyzed elimination reaction which results in the loss of monosaccharide units from the reducing end of the glycan (Merry et al., 2002). The mild reaction conditions for the DIB-mediated Hofmann rearrangement and the lack of off-target oxidation observed by Beckwith and others (Beckwith and Dyall, 1990) suggest that hypervalent iodine compounds could be more efficient glycan release agents. For the hypochlorite mediated glycan release, Song et al. (2016) (Song et al., 2016) have described a 20% loss of sample due to alkaline reducing end degradation (Supplementary Table S6) of their published manuscript (Song et al., 2016)). Due to mechanistic similarity, alkaline peeling is likely to occur with similar abundance for the hypochlorite-, oxone/bromide- and DCDMH- mediated glycan release reactions.

The analogy of the oxidative chemical glycan release and the Hofmann rearrangement suggests that all accessible carboxamides in a glycoprotein are being attacked during the release reaction. In fact, we were unable to detect clear bands for chemically deglycosylated protein samples on SDS-PAGE. Rather we detected smears indicative of severe random fragmentation of the protein backbone. This lack of selectivity is a major limitation of the described chemical methods for glycan release. Therefore, to avoid the massive fragmentation of the glycoprotein polypeptide moiety during chemical glycan release, a strategy to exploit the uniqueness of the glycan carboxamide is needed. Unlike this specialized type of carboxamide the peptide bond carboxamides can undergo a biuret reaction (Gornall et al., 1949). The addition of biuret reagent to the glycoprotein to protect the peptide bond carboxamides by metal complex formation prior to adding the Hofmann reactant may therefore be a possible route towards achieving a more selective chemical glycan release.

**FIGURE 10:**
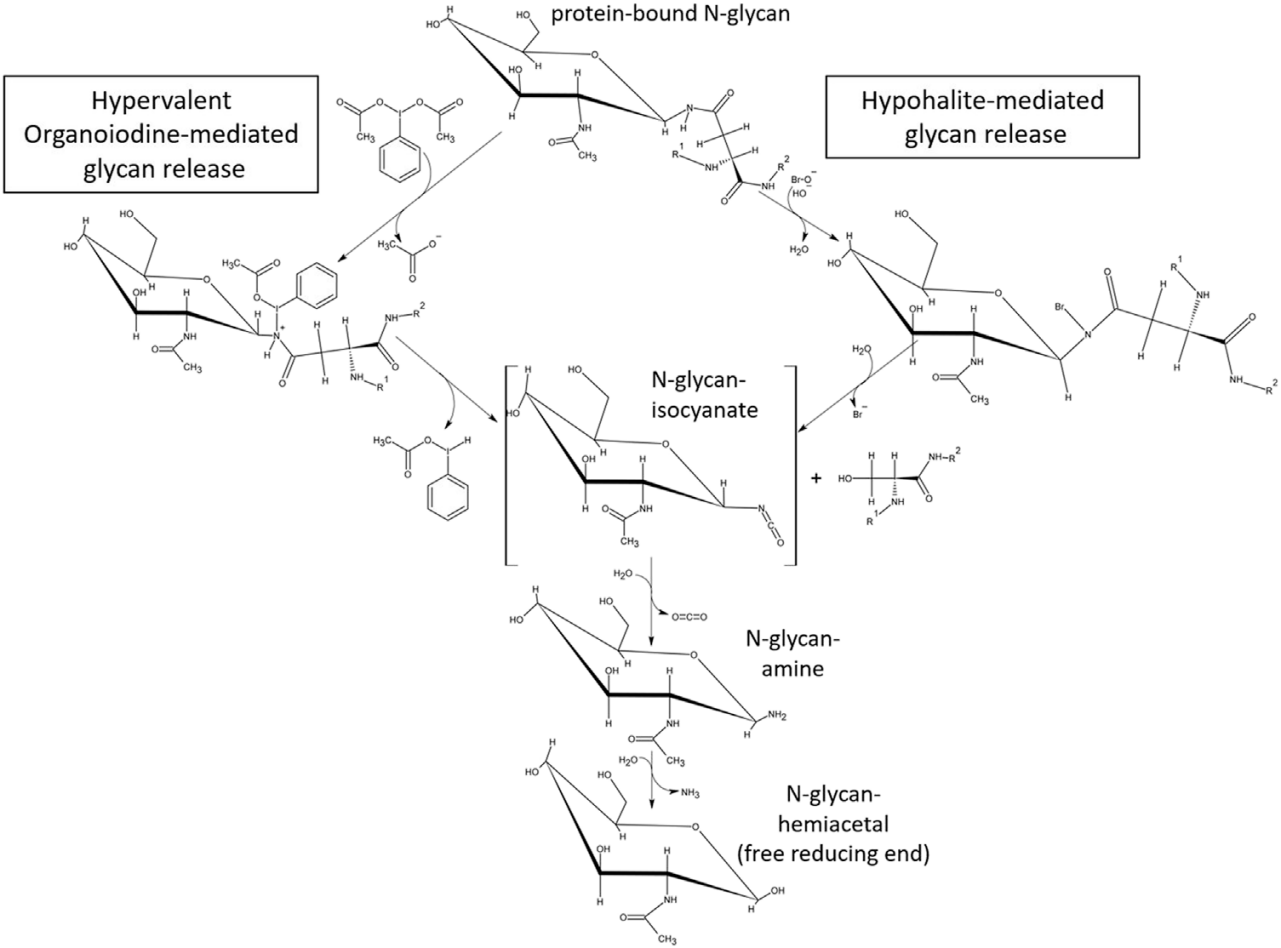
Proposed mechanism for glycan release by chemical reactants capable of forming an N-haloamide intermediate with glycoprotein-carboxamides followed by cleavage of the bond between the gamma-carbonyl- and beta-carbon of asparagine. The proposed mechanism for hypohalite mediated glycan release has been proposed previously by Song et al. (2016) and involves alkaline abstraction of a carboxamide N-H-proton, reaction of the resulting N-anion with the halogen atom of hypohalite, formation of the covalent N-haloamide and its rearrangement into an isocyanate. Hypervalent organoiodine (III) compounds (example shown: diacetoxy-iodobenzene, DIB) carrying a positive net atomic charge on the halogen form an intermediate N-iodoamide in the absence of alkaline conditions and the subsequent dissociation of the nitrogen-iodine bond under neutral pH conditions. In both cases the mechanism resembles the initial steps of the classical Hofmann carboxamide rearrangement.

While the release of glycans using hypervalent iodine-compounds still warrants optimization, the Oxone^®^/Bromide method has demonstrated its applicability with the three test glycoproteins—ovalbumin, IgG1 monoclonal antibody and RSVF. The glycan of human IgG1-Fc at position N297 is a bi-antennary complex-type structure composed of a core chitobiose and a branching trimannoside decorated with additional fucose, N-acetylglycosamine, galactose and N-acetyl-neuraminic acid monosaccharide units (Arnold et al., 2007). Our data show the classic pattern of predominant G0F, G1F and G2F glycan structures released by both PNGase F and Oxone^®^/Bromide methods (Figure 7F). The additional peaks detected at 19 and 27 min retention time in the HILIC-FLD spectrum of Oxone^®^/Bromide released mAb-Fc glycans may be due to an artifact of alkaline peeling in case of the peak at 19 min. The additional peak at 27 min retention time could be due to altered HILIC retention of Oxone^®^-oxidized glycans carrying additional aldehyde or carboxylate groups. Surprisingly, we discovered that the Oxone^®^/Bromide method was considerably more efficient in releasing N-glycans from a homogeneously glycosylated test protein with a core-F-type paucimannose glycan (Figures 8, 9). There are several potential explanations for this phenomenon. First, this could be due to a favorable amino acid sequence context surrounding each of the N-X-S/T-sequons of the RSVF glycoprotein. Yet, this seems to be rather unlikely considering the aggressive chemical glycan release mechanism and its lack of dependence on amino acid primary sequence. Second, the one factor that sets apart the RSVF from the other two glycoproteins tested is the presence of three occupied N-glycan sequons (Acc. No. EF566942) (Sandig et al., 2017). This suggests that the ratio of glycan to protein amount is the highest for RSVF compared to mAb or chicken ovalbumin: 518 amino acids/3 occupied N-glycan sequons, overall glycan/protein ratio = 0.58%; IgG1-mAb: 1334 aminoacids / two occupied N-glycan sequons, overall glycan/protein ratio = 0.15%; Chicken Ovalbumin: 386 amino acids/1 occupied N-Glycan sequon, overall glycan/protein ratio = 0.26%). Third, the small non-precipitable peptides resulting from inadvertent chemical cleavage of peptide bond carboxamides could compete for the glycan binding capacity of glycan enriching solid phases such as porous graphitic carbon and different HILIC phases for solid phase extraction of glycans, in turn affecting glycan yield. Fischler and Orlando have already discussed the possibility that the lower efficiency of chemical oxidative glycan release method may either be attributed to a low efficiency of the chemical mechanism or to glycan sample degradation (Fischler and Orlando, 2019). We would like to add a third possible explanation for this: a lowered recovery of chemically released glycans from the resulting matrix. Why any of these issues should be different between proteins is still unclear. The proposed mechanism of glycan release by chemical effectors of the Hofmann rearrangement mechanism is presented in Figure 10. The mechanism suggests that both hypohalites as well as hypervalent iodine compounds form an N-haloamide/N-iodine (III) species with the glycan N-linked γ-carboxamide of asparagine as an unstable intermediate that quickly disintegrates to form a metastable glycan-isocyanate and a carbenium-ion on the protein moiety. This carbenium-ion quickly reacts with hydroxide ions to form a serine residue in place of the asparagine side chain. When hypervalent iodine compounds are being employed as activating species in the Hofmann reaction, the attacked carboxamide reacts with the electrophilic organoiodine-atom to form an N-iodine(III) species. In contrast to hypohalite mediated reactions that initially form rather stable N-haloamides with carboxamides, the nitrogen–iodine bond is labile and therefore alkaline pH is not necessary for hypervalent organoiodine to effect the carboxamide rearrangement (Knochel, 2014).

In summary, the reported Oxone^®^/Bromide method allows for the robust and rapid release of protein-bound glycan-oligosaccharides by *in-situ* generated chemical effectors capable of forming an N-haloamide intermediate with glycoprotein-carboxamides. Future work will focus on investigating reactions with protected peptide bond carboxamides as well as glycan release reactions mediated by fluorinated hypervalent organoiodine compounds, mixtures of hypervalent organoiodine (III) and (V) reactants and catalytic amount of highly fluorinated additives.

## Materials and methods

### Materials

All chemicals and HPLC solvents were purchased from Merck KGaA (Darmstadt, Germany), Sigma Aldrich, Acros Organics (New Jersey, United States), Serva Electrophoresis GmbH, Carl Roth or Fisher Scientific. All electrophoresis reagents were purchased from SERVA Electrophoresis GmbH. Oxone^®^ was purchased from Medipool GmbH ((Cat.No: 591601 MP) Wendlingen, Germany) and prepared fresh before use. Hypochlorite solution was from W5 (2.8% NaClO) and diluted fresh before use. PNGase F was from NEB. Solid phase extraction Carbograph columns were purchased from Sigma Aldrich while the C18-based SPE mini-columns were from (Applichrom GmbH, Oranienburg). The monoclonal antibody from a research grade cell culture supernatant, was a kind gift from Dr. Volker Sandig, ProBioGen AG, Berlin, Germany. RSVF glycoprotein was produced as described in (Sandig et al., 2015).

### Preparation of carbohydrate hexose unit standards (GU units)

Carbohydrate standards were prepared from acid-hydrolyzed z previously (Goins and Cutler, 2000). Briefly, 100 mg of dextran (Cat.-No. 31398; molecular weight ∼200,000, Sigma) were suspended in 10 ml of 0.1 N HCl in a Duran test tube with screw cap and incubated at 100°C for 4 h. Then the solution was blow-dried in a Nitrogen evaporator and the dried dextran hydrolysate was stored at −20°C.

### Fluorescent labeling of N-glycans and standards

N-glycans were labeled with anthranilic acid (2-AA) by reductive amination. Briefly, to a 100 µl solution of glycans in water was added 50 µl 2-AA (48 mg/ml stock in DMSO) and 50 µl freshly prepared 1 M sodium cyanoborohydride (DMSO). The mix was acidified with acetic acid to a final concentration of 15 % (v/v). The reaction was incubated at 65 °C for 1 h and stopped with the addition of 50 µl 100% acetonitrile. 2-AA labelled Dextran standard was prepared by using 100 µl a stock solution 10 mg dextran hydrolysate/ml of dH_2_O in the above labelling reaction.

For labeling with 8-aminonaphthalene-1,3,6-trisulfonic acid (ANTS), the glycan sample or standard (1 mg dry weight) was dried completely. To each sample was then added 5 µl of 0.1 M ANTS (in 15% acetic acid in water) and 5 µl 1 M sodium cyanoborohydride (DMSO) and the reaction incubated at 37°C for 16 h. These reactions did not require the removal of excess label and therefore were directly analyzed by Blue Horizon horizontal fluorescence assisted carbohydrate electrophoresis (BHZ-FACE).

### Clean-up of 2-AA labelled glycan samples

The labeled glycans were separated from the excess label using a C18-SPE column (AppliChrom SPE-Kartuschen, Cat.-No: ACSPEC18EC750/6–150, C18 end-capped, 750 mg/6 ml, Applichrom GmbH, Oranienburg, Germany). Prior to use the C18 SPE column was activated and equilibrated by the following sequence of added mobile phases: activation by 3 ml of 100% acetonitrile, wash with 2 ml 80% (v/v) acetonitrile, wash with 2 ml 50% (v/v) acetonitrile, wash with 20% (v/v) acetonitrile /0.1% (v/v) formic acid. Then the 250 µl 2-AA labelled glycan sample was loaded onto the equilibrated C18-SPE column and the column was then eluted by passing 2 ml 20% (v/v) acetonitrile /0.1% (v/v) formic acid over the column with a flow rate of 0.5 ml/min. (air displacement positive pressure elution). The first 400 µl of the eluate contained polar impurities and were discarded. The second 600 µl eluate contained the majority of the 2-AA labelled glycans and were collected, the third 600 µl eluate fraction contained the bulk of uncoupled 2-AA excess label and were discarded. The second eluate fraction containing the 2-AA labelled glycans were dried in a SpeedVac (Savant SPD 1030, max. vacuum, 45°C, 2 h) and then resuspended in 30 µL of 65% ACN (v/v) for later analysis by RP-HPLC-FLD or HILIC-FLD.

### High-performance liquid chromatography

An Azura HPLC system from Knauer GmbH (Berlin, Germany) was used for HPLC analysis. To it was integrated a Jasco FP-1520 fluorescence detector and a column thermostat. A fluorescence excitation of 360 nm and emission of 420 nm was used for 2-anthranilic acid (2-AA)-labeled glycans and the dextran standard.

### Reversed Phase HPLC with fluorescence detection (RP-HPLC-FLD)

For RP-HPLC-analysis, 2-AA labelled glycan samples were resolved on a Knauer Azura System equipped with a Jasco 1520 fluorescence detector and an OTU TriKala C18 (105Å, 5 µm, 250 mm × 4.6 mm; or 105Å, 5 µm, 150 mm x 3.0 mm Applichrom GmbH, Oranienburg, Germany). 2-AA labelled glycan samples were resolved on RP-HPLC at 25°C column temperature by isocratic elution with 10% acetronitrile with 0.1% formic acid with flow rates ranging from 0.5–1.0 ml/min as indicated.

### Hydrophobic interaction liquid chromatography with fluorescence detection

2-AA labelled glycan samples were detected by HILIC-FLD using an Azura HPLC system (Knauer Wissenschaftliche Geräte GmbH, Berlin, Germany) coupled to a Jasco FP-1520 fluorescence detector and a column thermostate (JASCO International Co., Ltd., Tokio, Japan) equipped with an OTU-Amino column (105Å, 5 µm, 250 mm × 4.6 mm; Applichrom GmbH, Oranienburg, Germany). A fluorescence excitation of 360 nm and emission of 428 nm was used to detect 2-anthranilic acid(2-aminobenzoic acid, 2-AA)-labeled glycans and dextran standards. Samples were resolved on HILIC at 40°C column temperature by isocratic elution with 60% acetronitrile in 10 mM ammonium formate buffer pH 4.4 at a flow rate of 1.0 ml/min.

### Oxidative release of N-glycans using hypochlorite

The protocol was adapted from that described previously (Song et al., 2016). The following modifications were made: 100 ul glycoprotein (20 mg/ml) was mixed with 100 ul of a saturated borax solution. To this was added 200 ul of a 1% hypochlorite solution and incubated for 10 min at 37°C with shaking. The reaction was stopped by the addition of 20 ul formic acid, cooled briefly on ice and then centrifuged for 2 min at 10,000 g at room temperature. The supernatant was diluted 4–10-fold prior to purification on a carbograph SPE column. Briefly, the column was activated with 3 ml 100% acetonitrile and followed by a wash with 4 ml of distilled water. The diluted sample was loaded, washed briefly with 2 ml water and eluted in 0.5–1 ml 50% acetonitrile. The glycan sample was dried and resuspended in the requisite amount of water for labeling.

### Oxidative release of N-glycans using oxone^®^

0.0413 mg/ml Oxone^®^ was prepared fresh by the addition of water. As for hypochlorite, 100 µl glycoprotein (20 mg/ml) was mixed with 100 µl of a saturated borax solution and placed on ice. To this was then added 50 µl 5 M NaOH to adjust the pH to more alkaline values and 50 µl of a cold (at 4°C) saturated potassium bromide solution. Depending on the experiment, different concentrations of potassium bromide solutions were also used as indicated in the respective figures. After mixing gently, an equal volume of freshly prepared Oxone^®^ was added (300 µl) and the reaction mix immediately incubated at 37°C for 10 min. The reaction was stopped by the addition of 50 µl of saturated potassium metabisulfite and the proteins precipitated with trichloroacetic acid (TCA). After centrifugation for 2 min at 10,000 g at room temperature, the glycans were purified on a carbograph SPE as described above.

### Oxidative release of N-glycans using 1,3-dichloro-5,5-dimethylhydantoin

100 µl glycoprotein (20 mg/ml) was mixed with 100 µl of a saturated borax solution. To this was then added 200 µl of 67 mM DCDMH in 70% (v/v) Methanol and 10 µl saturated NaOH. The reaction mix was immediately incubated at 37°C for 5 min at 500rpm shaking speed. The reaction was stopped by the addition of 50 µl of saturated potassium metabisulfite and the proteins precipitated with 50 µl saturated TCA for 2 min on ice. After centrifugation for 2 min at 10,000 g at room temperature, the glycans were purified on a carbograph SPE as described above.

### Oxidative release of N-glycans using diacetoxy-Iodobenzene

100 ul glycoprotein (20 mg/ml) was mixed with 100 ul of a saturated borax solution. To this was added 42,5 mg of solid DIB and the mixture was then incubated for 72 h at 37°C with shaking. The reaction was stopped by precipitating the proteins with TCA on ice for 2 min. After centrifugation for 2 min at 10,000 g at room temperature, the glycans were purified on a carbograph SPE as described above.

### Enzymatic release of N-glycans

N-glycans were enzymatically released by PNGase F following a denaturation step as described in the vendors instructions (https://international.neb.com/protocols/2014/07/31/pngase-f-protocol).

### Preparation of BHZ-FACE glycan electrophoretic mobility standard

ANTS-labelled dextran partial hydrolysate was used as standard for assessing the electrophoretic mobility of different ANTS-labelled hexose units. This standard was prepared as described previously (Gois and Cutler, 2000). Briefly, 100 mg of dextran (Cat.-No. 31398; molecular weight ∼200,000, Sigma) were suspended in 10 ml of 0.1 N HCl and incubated at 100 °C for 4 h. Then the solution was blow-dried in a Nitrogen evaporator. The dried carbohydrate sample was suspended in 500 µl each of 0.2 M ANTS in acetic acid-water (3:17, vol/vol) and freshly made 1.0 M sodium cyanoborohydride in dimethyl sulfoxide and incubated at 37°C for 16 h. The samples were then dried in a fume hood under nitrogen at 45°C and stored at −20°C. Standard was dissolved in GlycoGel loading buffer (Serva) prior to use.

### Blue horizon horizontal FACE gel electrophoresis

A horizontal, ultrathin SERVA HPE™ GlycoGel (gel dimensions: 125 × 250 mm × 0.43 mm; Serva Electrophoresis GmbH) was placed on the chilled ceramic flatbed of an HPE™ BlueHorizon™ Flatbed Chamber (Serva Electrophoresis GmbH). Chilling temperature of the HPE™ Chiller unit (Serva) was pre-set to 5°C in advance and the ceramic plate was prepared with contact fluid (Serva) just prior to placing the gel. 2 µl of freshly prepared ANTS-labelled glycan samples were then directly loaded into the slots of the GlycoGel. The BluePower 3000 HPE™ Power Supply (Serva) was then programmed for the following runtime and power settings: U1 = 250 V; I1 = 25 mA; t = 30 min; U2 = 600 V; I2 = 40 mA; t = 5 min; U3 = 800 V; I3 = 50 mA; t = 50 min. After completion of the run, the BHZ-FACE gels were analyzed UV-illumination at UV 365 nm/ Filter 525 nm.

### Electrospray ionization mass spectrometry

The electrospray ionization mass spectra for 2-AA labeled glycan samples were recorded on a Varian-500 MS system equipped with an ESI source applying 5.0 kV on the MS inlet in positive ion-mode. For MS analysis, the sample peak was collected from HILIC runs with LC/MS-grade acetonitrile used for the mobile phase. The samples were infused into the ESI source at a flow rate of 5–10 μl/min. Drying gas (nitrogen) was set at 10 psi and at a temperature of 250°C. Nebulizing gas (nitrogen) was maintained at 35 psi. The flow rate of the damper gas (helium) was set at 0.8 ml/min. Spectra were acquired in a mass range of m/z 400–1,000. An external calibration was performed by infusing tuning mix solution (Varian/Agilent) at 10 μl/min.

## Data Availability

The raw data supporting the conclusion of this article will be made available by the authors, without undue reservation.

## Supplementary material

### Supplementary Figure S1

C18-HPLC-FLD profiles of N-glycan-2-AA conjugates released from ovalbumin (2 mg) with Oxone and potassium bromide. Variation of the applied concentration of sodium hydroxide solution (NaOH-Lsg.) (5 M, left; saturated, right), as well as the hydrolysis time at 37°C (5 min, top; 10 min, bottom) are shown. Note the optimum glycan yield achieved at 37°C, 10 min. incubation time in the presence of saturated NaOH.
