# Supplementary figures and images for: Release of protein N-glycans by effectors of a Hofmann carboxamide rearrangement
Source: Front Mol Biosci. 2022 Sep 12;9:983679. doi: 10.3389/fmolb.2022.983679 (PMC9512068; doi:10.3389/fmolb.2022.983679)

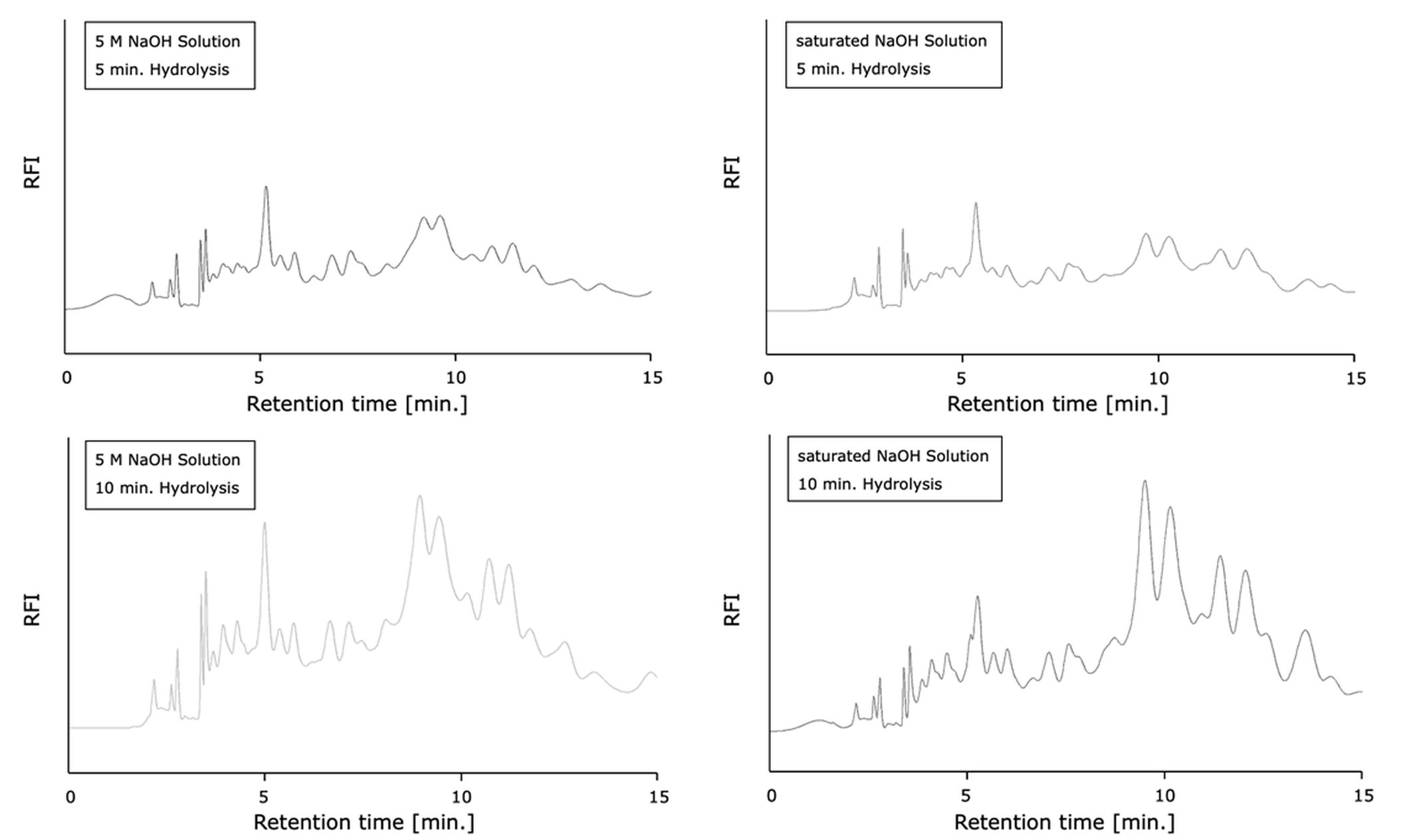

Supplement: Supplementary file 1 [file Image1.JPEG]
